# SARS-CoV-2 outbreaks in secondary school settings in the Netherlands during fall 2020; silent circulation

**DOI:** 10.1186/s12879-022-07904-3

**Published:** 2022-12-26

**Authors:** Lotte Jonker, Kimberly J. Linde, Marieke L. A. de Hoog, Robin Sprado, Robin C. Huisman, Richard Molenkamp, Bas B. Oude Munnink, Wietske Dohmen, Dick J. J. Heederik, Dirk Eggink, Matthijs R. A. Welkers, Harry Vennema, Pieter L. A. Fraaij, Marion P. G. Koopmans, Inge M. Wouters, Patricia C. J. L. Bruijning-Verhagen

**Affiliations:** 1 Julius Center for Health Sciences and Primary Care, UMC Utrecht, Utrecht University, Universiteitsweg 100, 3584 CG Utrecht, The Netherlands; 2 Institute for Risk Assessment Sciences, Utrecht University, Yalelaan 2, 3584 CM Utrecht, The Netherlands; 3 Department of Viroscience, Erasmus Medical Center, Dr. Molewaterplein 50, 3015 GD Rotterdam, The Netherlands; 4 Center for Infectious Disease Control, National Institute for Public Health and the Environment, Antonie Van Leeuwenhoeklaan 9, 3721 MA Bilthoven, The Netherlands; 5 Department of Medical Microbiology & Infection Prevention, Amsterdam University Medical Center, Meibergdreef 9, 1105AZ Amsterdam, The Netherlands; 6 Department of Pediatrics, Subdivision Infectious Diseases and Immunology, Erasmus Medical Center-Sophia Children’s Hospital, Wytemaweg 80, 3015 CN Rotterdam, The Netherlands

**Keywords:** SARS-CoV-2, Secondary schools, Transmission, Outbreak, Air levels

## Abstract

**Background:**

In fall 2020 when schools in the Netherlands operated under a limited set of COVID-19 measures, we conducted outbreaks studies in four secondary schools to gain insight in the level of school transmission and the role of SARS-CoV-2 transmission via air and surfaces.

**Methods:**

Outbreak studies were performed between 11 November and 15 December 2020 when the wild-type variant of SARS-CoV-2 was dominant. Clusters of SARS-CoV-2 infections within schools were identified through a prospective school surveillance study. All school contacts of cluster cases, irrespective of symptoms, were invited for PCR testing twice within 48 h and 4–7 days later. Combined NTS and saliva samples were collected at each time point along with data on recent exposure and symptoms. Surface and active air samples were collected in the school environment. All samples were PCR-tested and sequenced when possible.

**Results:**

Out of 263 sampled school contacts, 24 tested SARS-CoV-2 positive (secondary attack rate 9.1%), of which 62% remained asymptomatic and 42% had a weakly positive test result. Phylogenetic analysis on 12 subjects from 2 schools indicated a cluster of 8 and 2 secondary cases, respectively, but also other distinct strains within outbreaks. Of 51 collected air and 53 surface samples, none were SARS-CoV-2 positive.

**Conclusion:**

Our study confirmed within school SARS-CoV-2 transmission and substantial silent circulation, but also multiple introductions in some cases. Absence of air or surface contamination suggests environmental contamination is not widespread during school outbreaks.

**Supplementary Information:**

The online version contains supplementary material available at 10.1186/s12879-022-07904-3.

## Introduction

The role of schools in the transmission of Severe Acute Respiratory Syndrome Corona Virus 2 (SARS-CoV-2) has been the focus of continuous debate throughout the COVID-19 pandemic. Throughout most of the first year of the pandemic, the Dutch government implemented only a limited set of COVID-19 preventive measures in educational settings to minimise educational disruption. Between November and December 2020, we conducted a series of four detailed outbreak investigations in schools that reported clusters of SARS-CoV-2 infections. At the time, all primary and secondary schools were open and had full occupancy. There was little prior immunity against SARS-CoV-2, vaccines were not yet available and the wild-type variant with the D614G mutation was dominant at the time of the study.

The aim of the outbreak investigations was to provide a more detailed analysis on transmission risk in secondary school settings under the prevailing community incidence and COVID-19 mitigation policy, and to gain insight into the potential role of SARS-CoV-2 transmission via air and surfaces in schools.

## Methods

The outbreak investigations were part of a prospective school surveillance study that evaluates the interactions between indoor air quality, ventilation, environmental SARS-CoV-2 contamination and transmission. For this prospective cohort study, schools were selected in collaboration with the Dutch counsel for secondary education based on their size, educational provision and geographical location to create a representative sample of the Dutch landscape of secondary schools. At the time the outbreak investigations were performed, physical distancing (> 1.5 m) in schools was implemented for staff–staff and staff–student interactions, but not required in or outside school among children below 18 years. Only symptomatic individuals could get tested at municipal health facilities. Students and teachers who tested positive for SARS-CoV-2 were asked to self-isolate at home. Municipal testing services conducted contact tracing for each positive case. Close contacts were defined as individuals with exposure of > 15 min at < 1.5 m distance to a SARS-COV-2 infected individual and requested to quarantine. In case of school contacts, an exemption existed on prevailing contract tracing and quarantine rules at the time. Close school contacts were not actively approached by contact tracing teams and exempt from quarantine and could continue to attend school unless they developed symptoms. From December 2020 onwards, mask mandates were in place for students and staff during movement. Seated students and staff did not wear masks. Schools were recommended to increase hand hygiene and the degree of ventilation, plastic shields were installed on teacher desks and all school-based extracurricular activities were cancelled. Availability of SARS-CoV2 testing was expanded to asymptomatic close contacts during the study period (1 December 2020). Other national and school-initiated COVID-19 measures in place at the time, are described in the Additional file 1: Methods and Table S1.

All schools participating in the prospective study kept daily logs of SARS-CoV-2 infections that were identified at the municipal testing service and reported to the school. This included students and staff. Schools subsequently assessed whether there were possible epidemiological links between the reported cases based on student–teacher group compositions and timetables. An epidemiological link was defined as two or more reported cases who shared (class) rooms for at least two course hours in the recent 14 days. Schools notified the study team if a cluster was identified, which was defined as three or more reported cases in the same school within 2 weeks of whom at least two cases had an epidemiological link. The study team was available during school days to support schools in the assessment of epidemiological links between reported cases and school clusters. If a clusters was confirmed and the most recent reported case belonging to the cluster had been attending school in the 48 h prior to symptom onset or PCR test result an outbreak investigation was initiated among their school contacts who received onsite education.

### Outbreak study

A school visit was scheduled within 48 h after the notification of a cluster to sample participating school contacts. School contacts were defined as students and staff who had shared a (class)room for at least two course hours in the 2 days preceding symptom onset in the index case or, if this was unknown, the date of a positive test. A sampling location at the school was set-up where participants could self-collect a combined mid-turbinate NTS sample and a saliva sample under direct supervision of trained study staff after instructions had been provided. For participants who were not present at school, samples were self-collected under supervision of study staff at their home address. Participants also completed a questionnaire including basic demographics, recent COVID-19 infection within the household and other recent exposure to SARS-CoV-2 infected individuals (other than school index case or household), prior infection and presence of COVID-19 like symptoms. A second sampling visit was scheduled after 4–7 days, depending on the weekends, for a follow-up NTS and saliva sample from each participant, along with a follow-up questionnaire on recent exposure, symptoms and whether household members tested positive since the previous visit. For a schematic overview of the study design see Additional file 2: Figure S1.

All samples were transported to the laboratory the same day and participants were notified about the results of the PCR test within 48 h. Positive results were followed by self-isolation as per national policy. Saliva samples were stored at – 80 °C and analysed at a later time.

At the first visit, extensive air and surface sampling took place in school buildings (see Additional file 1: Methods and Additional file 2: Figure S1). Briefly, air samples and surface swab samples were collected at three locations: (1) classrooms attended by students previously in contact with the index cases, (2) the teachers’ lounge and (3) the school cafeteria area. At each location, air sampling consisted of twice a 6-h filtration-based sample, once a 6-h cyclone-based sample and once a 1.5-h impingement-sample (school cafeteria and teachers’ lounge only). Surface swab samples of high- and low-touch surface areas were collected as described previously [1]. A total of five samples were taken in each of the areas above and, when possible, from the classroom where index case teachers were located prior to self-isolation. Field blank samples were collected every other outbreak measurement for air samples, and each outbreak for surface swab samples. Samples were sent to the laboratory at 4 °C and processed within 24-h.

### Sample analysis

Detailed methods are described in the Additional file 1: Methods. NTS were collected in tubes containing virus transport medium and total nucleic acid was extracted as described [2]. Oral fluid was collected using the ORACOL S10 saliva collection system (Malvern Medical Developments). Total nucleic acid was extracted using MagNApure 96 (Roche LifeScience) small volume total nucleic acid kit. RT-qPCR was performed as described previously [2], with some modifications on the primers and probe of the RdRP-gene (see Additional file 1: Methods). From the environmental samples, RNA was isolated using an in-house method as described before [3]. Samples were tested with a SARS-CoV-2 Real time polymerase chain reaction (RT-PCR), targeting the E gene of SARS-CoV-2 [2].

Sequencing of NTS RT-PCR positive samples with Ct-values < 32 was performed using an amplicon-based approach as described [4]. For RT-PCR positive saliva samples, sequencing was performed using the Nanopore protocol [5, 6] with several modifications (see Additional file 1: Methods for details).

A secondary case was defined as a school contact participating in the study and testing positive by RT-PCR in at least one of the samples collected during initial or follow-up visits. According to standardised local lab protocols a Ct-value cut-off for sample positivity was set < 40 for both targets or at < 33 if only one target was positive. Samples were defined as ‘weakly positive’ if the Ct-value for a single target was between 33–40 and negative for the other target.

### Statistical analysis

SARS-CoV-2 incidence rates per schools were calculated by dividing the number of reported infections by the total number of students and staff members. Next, the secondary attack rate (SAR) per cluster was determined by dividing the secondary cases by the total number of participants in the outbreak investigation who underwent SARS-CoV-2 testing, both overall and stratified for teachers and students. Case characteristics, school attendance, presence of symptoms, onward household transmission and time since exposure were graphically displayed for all secondary cases. Possible onward household transmission was defined as a household member testing positive after the participant’s first positive test. All successfully sequenced NTS and saliva samples from both test rounds were combined in a phylogenetic reconstruction and are depicted per sample type. If the sequence was available from both test rounds, only the sequence of first round was included in the tree. We also included human sequences from the municipalities of the respective schools as background data, which was retrieved from GISAID. SPSS version 26.0.0.1 (IBM), and R version 4.0.3 (R core team) was used for data management and statistical analysis.

## Results

Between 11 November and 15 December 2020, we conducted four outbreak investigations. The overall weekly incidence of SARS-CoV-2 across the four participating schools during the study period varied between 299–820 per 100,000 students and staff members, while the weekly incidence in het Dutch population during the same period varied between 184 and 430 per 100,000 inhabitants (Table 1) [7, 8].

**Table 1:** School characteristics and weekly incidence of the four schools participating in the outbreak study (n = 4)

School	Size	Weekly number of SARS-CoV-2 infections (incidence per 100,000)^a^						
		week 45	week 46	week 47	week 48	week 49	week 50	week 51
A	> 2000	11 (530)	**7 (337)**	16 (771)	17 (819)	12 (578)	5 (241)	Missing
B	700–1000	NA	NA	**2 (262)**	4 (524)	3 (393)	0	3 (393)
C	1500–2000	2 (118)	3 (176)	**6 (352)**	10 (588)	5 (294)	4 (235)	3 (176)
D	500–700	0	1 (173)	0	11 (1900)^b^	5 (864)	**10 (1727)**	8 (1382)
Mean population incidence per 100,000		232	204	198	184	237	317	430

*NA* Not applicable

Highlighted in bold are the school and week where an outbreak investigation was initiated. School B enrolled in week 47 in the study. According to definition, clusters could evolve over > 1 week. Therefore the cases in bold will not add up with the number of index cases within the different clusters

^a^Students and staff combined. Numbers based on daily logs reported by the schools and excluding additional asymptomatic cases detected during our outbreak studies

^b^10/11 were from the same class. Considering the extent of this outbreak, the school decided to send the entire class home and convert to online education. Consequently, no outbreak investigation could be performed

Of the 148 reported cases, 27 index cases belonged to a cluster, including 6 staff members and 21 students. The total number of identified clusters was 6 and cluster size varied between 3 and 12 cases (Additional file 3: Table S2). However, in one cluster, the school did not report to the study team within the 48 h window and no outbreak investigation was initiated. In another cluster, the cluster involved already 10 students upon identification and the school therefore decided to quarantine the entire student group and convert to online education. Hence, no outbreak investigation could be conducted among school contacts.

**Fig. 1:**
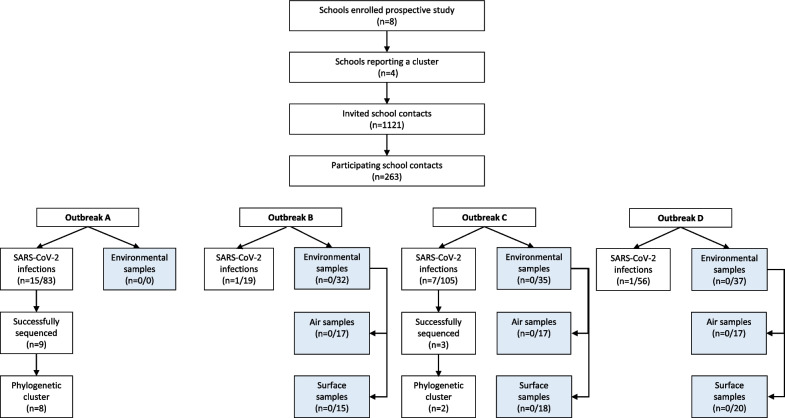
Overview of participating schools and school contacts. Number of SARS-CoV-2 infections among school contacts and positive air and surface samples are depicted per outbreak study. No environmental samples were taken during outbreak A

A total of 1121 school contacts were invited to participate across four clusters (Fig. 1). Cluster C further developed during the outbreak study and additional school contacts were therefore invited during the second sample round. While setting-up the study in school D, several additional cases were reported by the municipal health services and these cases were included as additional index cases belonging to the same cluster and resulted in additional school contacts being approached for participation. The number of staff exposed to a teacher index case could not be determined reliably as staff–staff contacts occur mostly in the teachers’ lounge. Therefore, all staff members were invited, but informed that they should only participate if they had been in close contact with any of the index cases. In total, 263 school contacts participated, including 93 staff members (Fig. 1). The participation rate was 10.6% to 41.8% among staff, and 7.5% to 56.1% among students (Additional file 3: Table S3). Eighteen subjects participated only in the first test round. In total 508 paired NTS and saliva samples were collected.

### Secondary cases

In total, 24 school contacts (9.1%) tested positive by RT-PCR in at least one sample (Fig. 1). Of these, 10 (41.6%) had a weakly positive PCR result. The SAR by cluster varied between 1.8–18.1% and was generally higher among students compared to staff (Additional file 3: Table S3). Table 2 describes the temporal pattern of exposure, school attendance, SARS-CoV-2 PCR results and symptoms among secondary cases. Out of the 21 secondary cases of whom we obtained symptom data, only eight (38.1%) were symptomatic at any time during follow-up. In four of these participants, the symptoms were present at the time of first sample collection. Notably, three of them attended school while symptomatic. The other four subjects developed symptoms 1 to 3 days after the positive PCR result. Out of the 13 asymptomatic individuals, 8 (62%) were weakly positive, while none of the symptomatic individuals were weakly positive. Onwards SARS-CoV-2 infection among household members was reported for 2 out of 21 (10%). One of the participants from school A was already quarantined because of a positive test of a household member, while a student from cluster C reported a family member who tested positive the same day.

**Table 2:**
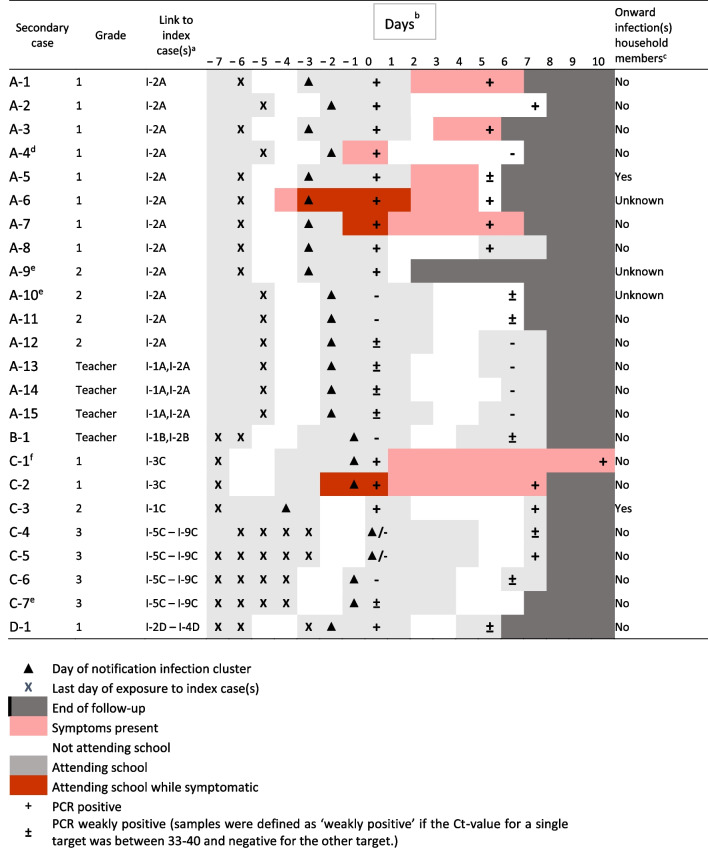
Characteristics of secondary cases belonging to each of the SARS-CoV-2 school clusters (n = 24)

^a^Based on school contacts who shared (class) rooms for at least two course hours in the recent 14 days

^b^Day 0 is set at the date of the first sample for each subject. Some sampling rounds were spread over 2 days, therefore days between exposure to index and first sample may vary per subject

^c^Defined as infections in household members detected after day 0

^d^A household member of this case tested positive in the 2 weeks before study initiation

^e^No symptom data available

^f^Household member tested positive but within 24 h after the student’s result

**Fig. 2:**
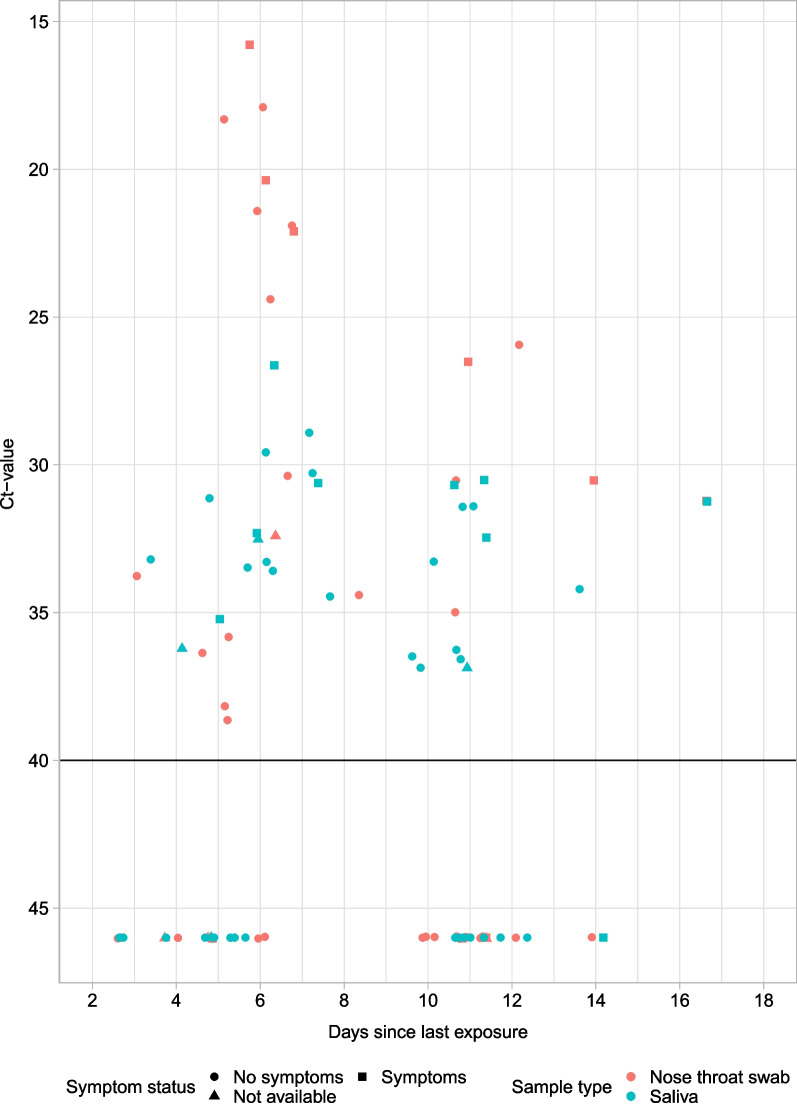
Ct-values by day since last exposure for nose throat swabs and saliva (n = 46). Ct-values by day since last exposure for nose throat swabs (orange) and saliva (blue). All samples from subjects with at least one positive results are included**.** Negative results are displayed as Ct-value > 45

In total 46 paired specimen samples from secondary cases were available (two cases did not participate in the second test round). Discrepancies in test results between the two, self-collected, specimens were observed in 19 out of 46 pairs (Additional file 3: Table S4). Eight of the 24 secondary cases tested positive only in saliva and five only in NTS. Testing of a second NTS and saliva sample after 3–7 days increased the detection rate by 33%. Lowest Ct-values were detected for samples taken between day 5 and 8 since last exposure and in symptomatic individuals (Fig. 2).

**Fig. 3:**
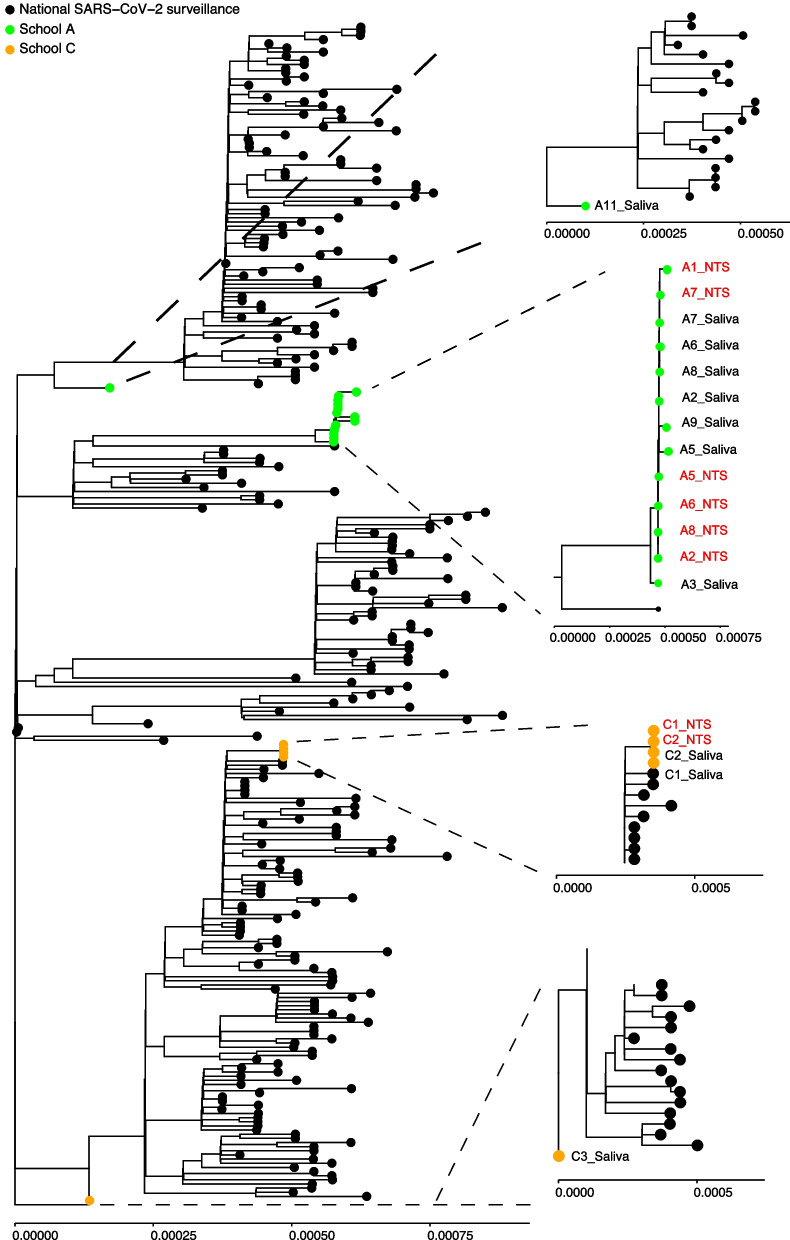
Sequencing data of RT-PCR positive saliva and nose throat swabs within school A and C. NTS Nose throat swab. Both test rounds are included in the figure. If sequencing data was available for both test rounds, only the sequence of the first round is included in the phylogenetic tree. Identification of NTS samples are depicted in red and for saliva samples in black

In school A and C multiple secondary cases were present which allowed to investigate confirmation of a cluster of infection by phylogenetic analysis (Fig. 3). A total of 12 individuals were successfully sequenced of which 9 and 3 originated from school A and C respectively.

In school A, the SARS-CoV-2 RNA sequences originating from eight participants formed a cluster. Demographic data revealed that all participants from this cluster were students and had only been in contact with each other during school hours. They came from two different classes who had been in contact with the same index case teacher. All participants within the cluster did not report any household members or other contacts testing positive before their positive test result. In addition, in one student a divergent strain was identified, indicating a separate introduction.

In school C, four SARS-CoV-2 sequences from two students phylogenetically clustered. They were classmates and only had been in contact with each other and the student index case during classes. One of the students reported a household member who tested positive the same day. For this school, also a phylogenetically distinct strain within the outbreak was identified, again showing a second, independent introduction. These results clearly show genetically linked transmission clusters, but also show other distinct strains within outbreaks, indicative of multiple introductions.

### Environmental sampling of air and surfaces

In total 104 environmental samples were collected from clusters B, C and D at the start of each investigation. All the 51 collected air and 53 surface swab samples tested negative for SARS-CoV-2. All field blank samples tested negative in PCR. For a complete overview of sample locations and results, see Additional file 3: Results and Table S5.

## Discussion

This detailed outbreak investigation among school contacts of four SARS-CoV-2 clusters using repeated PCR testing of two complementary specimens yielded a positivity rate of 9.1% with the majority of cases (61.9%) being asymptomatic. The sequence results together with detailed contact data indicate the presence of a cluster within school A. Likewise, in school C the sequencing and contact tracing data suggest that two individuals within the same class had infected each other. Remarkably, three out of the eight symptomatic individuals were present at school, implying that the message to stay at home and test when having symptoms was not always properly followed-up. Combined, these observations indicate that silent transmission of SARS-CoV-2 in secondary schools may occur. Yet, no SARS-CoV-2 contamination was detected in any of the air and/or surface samples collected from the schools during the period of the outbreak, suggesting that environmental contamination was not widespread.

Reported SAR values from other school outbreak investigations from the same period that used comparable methodology ranged between 0.0 and 6.5% [9–18]. The majority of the secondary cases in these studies were asymptomatic (47.8–66.6%), in line with our observations [10, 17]. Studies that used symptom based testing alone reported no secondary cases [19, 20]. In comparison, in our study symptom-based testing by means of a single NTS as was the policy at the time, would only have identified six cases yielding a SAR of 2.3% compared to 9.1%. In Israel, a large outbreak was reported in a school 10 days after reopening. Testing of the complete school community revealed an attack rate of 13.2% in students and 16.6% in staff members of which 47.8% were asymptomatic [21].

Combined, these results illustrate the importance of the applied testing strategy in estimating outbreak sizes in (school) populations, where silent circulation of SARS-CoV-2 infections can be easily missed. In our study, we increased our detection rate by repeated testing after 7 days and combining NTS and saliva samples, which could partially explain our higher detection rate compared to other outbreak studies. It should be noted that 10 individuals were weakly positive and for mitigating transmission (early) detection of such cases may be less important.

Nevertheless, the role of asymptomatic and pre-symptomatic infections in propagating the COVID-19 epidemic is now widely acknowledged, in particular among school students because of their more intense contact patterns. In our study, possible onward transmission to household members was suggested for 10% of the secondary cases for whom this data was available.

Although we found evidence of SARS-CoV-2 transmission within secondary schools, the lack of detectable SARS-CoV-2 RNA in collected air and surface samples suggests that major environmental contamination was uncommon in schools under the prevailing conditions at the time of the study. This is in contrast with findings from previous outbreak investigations conducted at mink farms and nursing homes, where similar sampling technologies were applied [1, 22]. In these studies, several air samples collected in COVID-19 infected mink farms, and a high percentage of both air and surface swab samples collected in rooms in nursing homes with SARS-CoV-2 positive patients [1, 22]. A previous study in London also found limited evidence of SARS-CoV-2 contamination in school environments [18]. Only in a minority (< 2–5%) of surface swab samples taken in both the classrooms of index cases and the washrooms, low amounts of viral RNA could be detected, some of which were collected before deep cleaning took place. In this study only 1/68 (1.5%) of the air samples was positive for SARS-CoV-2 [18]. Several factors could explain our negative results. First, schools implemented various measures to increase (hand) hygiene and prevented social gatherings. Second, schools increased their ventilation regimes by opening doors and windows and installing new mechanical ventilation systems. Although, the effect of these interventions on SARS-CoV-2 transmission is still unclear. Third, in the nursing home and mink farm studies samples were collected in the vicinity of acute phase shedding SARS-CoV-2 patients or minks. This is in contrast with the secondary schools, where the known cases were isolated at home. It was not possible to determine whether one or more of the SARS-CoV-2 infections identified through the intensified screening were present in the room during air sampling due to restrictions associated with privacy regulations. Moreover, most infected students and teachers were asymptomatic or pauci-symptomatic which is known to be associated with lower infectiousness [23]. Lastly, SARS-CoV-2 spread and transmission is suggested to be a more local phenomenon, suggesting direct droplet contact and/or close range aerosol (up to several meters) as the dominant route of transmission in the school environment [22, 24].

The major strength of this study is that we collected a large amount of data in school contacts (e.g. sequencing, symptom onset and recent exposure), irrespective of symptoms, which provided an opportunity to obtain extensive virological and contact tracing information from the subjects. Furthermore, we increased our detection rate by combining specimens and testing twice. Lastly, the combined sampling of the environment and school contacts facilitated identification of transmission mechanisms within secondary schools. However, some limitations need to be addressed: First, we investigated only four outbreaks and observed a high variability in SAR between clusters, reflecting the stochasticity in our data. Second, the low participation rate among contacts may have resulted in under- or overestimation of the SAR due to selection bias. Third, we only invited students to participate if they shared a classroom with the index case. Consequently, we may have missed secondary cases among other school contacts with whom the index case spent time during breaks. Fourth, we cannot conclude that the observed SAR solely reflects school transmission rates, because sequencing of samples was incomplete for secondary cases and not available for index cases. The SAR may therefore have been somewhat inflated by simultaneous unrelated introductions. However, apart from two participants in our study, no other participants reported contact with a known case outside the cluster. Fifth, cluster detection may have been incomplete because of the limited testing policy at the time and because schools may have been incomplete in their reporting of clusters. To minimize this underreporting, schools could contact the study team daily throughout the study to discuss the situation in their school and to assess whether criteria for an epidemiological link and cluster were met. Last, the outbreak investigation was performed during the pre-alpha period when school aged children were not vaccinated and there was less prior immunity in the population. Therefore, the results should be interpreted in the context of the epidemiological situation at the time.

## Conclusion

In conclusion, our study confirmed within school SARS-CoV-2 transmission, but also multiple introductions and substantial silent circulation at a time with limited COVID-19 prevention measures in secondary school settings and minimal prior immunity. Absence of widespread air or surface contamination suggests transmission may have occurred most likely via direct route or close range aerosol transmission route. Repeated testing is complementary and therefore recommended when complete case detection is desired. These insights can contribute to the discussion on the role of secondary schools in the transmission of SARS-CoV-2 and how to improve future outbreak studies.

## Supplementary Information

**Additional file 1.** Additional methods

**Additional file 2: Figure S1.** Overview of study procedures during an outbreak investigation at a school. Environmental samples are depicted in blue and human samples are depicted in white

**Additional file 3.** Additional results

## Data Availability

The sequence data supporting the conclusions of this article are available in the GISAID repository (www.gisaid.org; Accession ID: EPI_ISL_722426-722430, EPI_ISL_722432, EPI_ISL_722290, EPI_ISL_722334). Raw data are available upon reasonable request from the corresponding author.

## Abbreviations

RT-PCR: Real time polymerase chain reaction
SARS-CoV-2: Severe Acute Respiratory Syndrome Corona Virus 2
SAR: Secondary attack rate
WMO: Medical Research Involving Human Subjects Act
