# Supplementary figures and images for: SARS-CoV-2 outbreaks in secondary school settings in the Netherlands during fall 2020; silent circulation
Source: BMC Infect Dis. 2022 Dec 26;22:960. doi: 10.1186/s12879-022-07904-3 (PMC9791966; doi:10.1186/s12879-022-07904-3)

**Figure S1. Schematic overview of the study design**

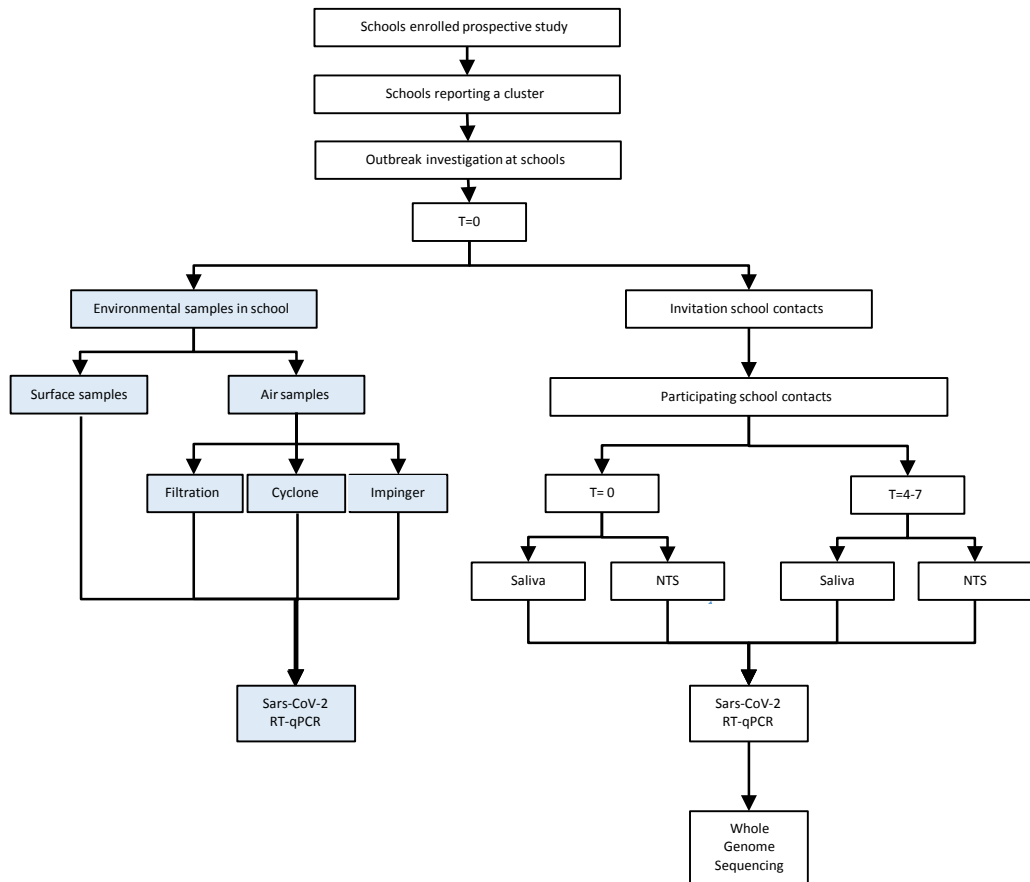

Supplement: Supplementary file 2 — Additional file 2: Figure S1. Overview of study procedures during an outbreak investigation at a school. Environmental samples are depicted in blue and human samples are depicted in white [file 12879_2022_7904_MOESM2_ESM.pdf]
