# Supplementary material for: SARS-CoV-2 outbreaks in secondary school settings in the Netherlands during fall 2020; silent circulation
Source: BMC Infect Dis. 2022 Dec 26;22:960. doi: 10.1186/s12879-022-07904-3 (PMC9791966; doi:10.1186/s12879-022-07904-3)
Supplement: Supplementary file 3 — Additional file 3. Additional results [file 12879_2022_7904_MOESM3_ESM.docx]

## **Supplementary results**

**Table S2. Characteristics of index cases**

| **Index case** | **Staff/student** | **Days since contact^a^** | **Days at school^b^** | **Any symptoms** |
| --- | --- | --- | --- | --- |
| I-1A | Staff | 0 | 1 | Y |
| I-2A | Staff | 0 | 1 | Y |
| I-3A | Student | 2 | 1 | Y |
| I-1B | Staff | 6 | 2 | Y |
| I-2B | Staff | 7 | 1 | Y |
| I-3B | Student | 0 | 1 | Y |
| I-1C | Staff | 1 | 2 | Y |
| I-2C | Student | 0 | 1 | Y |
| I-3C | Student | 0 | 1 | Y |
| I-4C | Student | 0 | 1 | Y |
| I-5C | Student | 11 | 2 | Unknown |
| I-6C | Student | 10 | 2 | Y |
| I-7C | Student | Unkown | Unknown | Y |
| I-8C | Student | 9 | 2 | Y |
| I-9C | Student | 13 | 2 | Unknown |
| I-1D | Student | 2 | 3 | Unknown |
| I-2D | Student | 0 | 2 | Y |
| I-3D | Student | 3 | 0 | N |
| I-4D | Student | 4 | 8 | Y |
| I-5D | Staff | 6 | 0 | Y |
| I-6D | Student | 9 | 1 | Y |
| I-7D | Student | 8 | 3 | Y |
| I-8D | Student | 7 | 1 | Unknown |
| I-9D | Student | 9 | 1 | Y |
| I-10D | Student | 9 | 1 | Y |
| I-11D | Student | 9 | Unknown | Unknown |
| I-12D | Student | 10 | 3 | Y |

^a^ Number of days between positive test in current case and first index case in the cluster

^b^ Days at school; number of days present at school during the presumed infectious period (i.e. from two days before date of symptom onset or, if unknown/asymptomatic, the test date)

In cluster C we were already sampling participants for the second test round while additional 6 cases were reported to the school. These 6 cases were therefore all considered part of the cluster and qualified as index cases according to definition. A similar situation occurred in cluster D the team was informed when three cases had just been detected, but by the time we initiated our study 24 hrs later this number had already increased to 12.

**Table S3. Details of outbreak investigations**

| **Cluster** |  | **Invited** | **Participated** | **Median age participants (IQR)** | **Percentage male** | **SARS-CoV-2 positive** | **Positivity rate** |
| --- | --- | --- | --- | --- | --- | --- | --- |
| A | staff | 184 | 28 (15.2%) | 33.7 (29.8-55.1) | 46.4% | 3 | 10.7% |
|  | students | 98 | 55 (56.1%) | 13.0 (12.4-13.6) | 20.0% | 12 | 21.8% |
| B | staff | 104 | 11 (10.6%) | 40.1 (31.5-48.3) | 63.6% | 1 | 9.1% |
|  | students | 106 | 8 (7.5%) | 12.7 (12.3-13.6) | 50.0% | 0 | 0.0% |
| C | staff | 182 | 26 (14.3%) | 41.9 (36.2-57.4) | 50.0% | 0 | 0.0% |
|  | students | 241 | 79 (32.8%) | 15.3 (14.8-15.7) | 44.3% | 7 | 8.9% |
| D | staff | 67 | 28 (41.8%) | 49.3 (40.5-58.8) | 35.7% | 0 | 0.0% |
|  | students | 139 | 28 (20.1%) | 12.9 (12.7-14.7) | 57.1% | 1 | 3.6% |

**Table S4. Ct-values of different specimens and sample time points among secondary cases (n=24)**

| **First sampling round** | | | | | | **Second sampling round** | | | | |
| --- | --- | --- | --- | --- | --- | --- | --- | --- | --- | --- |
| **Secondary case** | **Days since last exposure** | **Nose throat swab**  **Ct-value** | | **Saliva Ct-value** | | **Days since last exposure** | **Nose throat swab**  **Ct-value** | | **Saliva Ct-value** | |
|  |  | **RdRP-gen** | **E-gen** | **RdRP-gen** | **E-gen** |  | **RdRP-gen** | **E-gen** | **RdRP-gen** | **E-gen** |
| A-1 | 6 | **27.30** | **24.40** | **33.59** | **34.49** | 11 | **26.50** | **26.50** | **32.46** | **35.85** |
| A-2 | 5 | **20.80** | **18.30** | **31.17** | **31.13** | 12 | **28.90** | **25.90** | Neg | Neg |
| A-3 | 6 | Neg | Neg | **33.29** | **35.28** | 11 | Neg | Neg | **30.51** | **31.52** |
| A-4 | 5 | Neg | Neg | **35.22** | **35.22** | 11 | Neg | Neg | Neg | Neg |
| A-5^a^ | 6 | **23.90** | **21.40** | **33.51** | **33.48** | 11 | Neg | Neg | Neg | **36.26** |
| A-6 | 6 | **22.30** | **20.40** | **27.02** | **26.63** | 11 | Neg | **30.50** | **31.89** | **31.42** |
| A-7 | 6 | **19.60** | **15.80** | **32.95** | **32.31** | 11 | Neg | Neg | **30.69** | **31.70** |
| A-8 | 6 | **20.60** | **17.90** | **29.95** | **29.58** | 11 | Neg | **35.00** | **31.40** | **32.76** |
| A-9^a^ | 6 | Neg | **32.40** | **32.52** | **33.02** | *NA* | - | *-* | *-* | *-* |
| A-10^a^ | 5 | Neg | Neg | Neg | Neg | 11 | Neg | Neg | Neg | **36.88** |
| A-11 | 5 | Neg | Neg | Neg | Neg | 11 | Neg | Neg | Neg | **36.58** |
| A-12 | 5 | Neg | **35.80** | Neg | Neg | 11 | Neg | Neg | Neg | Neg |
| A-13 | 5 | Neg | **38.20** | Neg | Neg | 11 | Neg | Neg | Neg | Neg |
| A-14 | 5 | Neg | **38.60** | Neg | Neg | 11 | Neg | Neg | Neg | Neg |
| A-15 | 5 | Neg | **36.40** | Neg | Neg | 11 | Neg | Neg | Neg | Neg |
| B-1 | 6 | Neg | Neg | Neg | Neg | 12 | Neg | **36.60** | Neg | Neg |
| C-1 | 7 | **24.30** | **21.90** | **30.76** | **30.28** | 17 | **33.70** | **31.20** | **31.24** | **31.51** |
| C-2 | 7 | **24.50** | **22.10** | **30.62** | **31.93** | 14 | **31.60** | **30.50** | Neg | Neg |
| C-3 | 7 | **32.10** | **30.40** | **28.91** | **29.18** | 14 | Neg | Neg | **34.21** | **34.84** |
| C-4 | 3 | Neg | Neg | Neg | Neg | 10 | Neg | Neg | Neg | **36.48** |
| C-5 | 3 | Neg | Neg | Neg | Neg | 10 | Neg | Neg | **33.28** | **35.31** |
| C-6 | 4 | Neg | Neg | Neg | Neg | 10 | Neg | Neg | Neg | **36.87** |
| C-7^a^ | 4 | Neg | Neg | Neg | **36.22** | *NA* | *-* | *-* | *-* | *-* |
| D-1 | 3-7 | Neg | **33.80** | **33.20** | **33.52** | 8-12 | Neg | **34.40** | Neg | **34.46** |

^a^ Case had symptoms between day 3-5, but not on test days

^b^ No symptom data available

Highlighted in red are samples from individuals who reported symptoms at the day of the test

**Table S5. Descriptives and results of detection of SARS-CoV-2 RNA in air and surface samples in the school environment during outbreak measurements.**

|  |  | **Air measurements** | | | | | **Surface measurements** |
| --- | --- | --- | --- | --- | --- | --- | --- |
| **Outbreak** | **Location** | **CIS** | **NIOSH** | | | **Impinger** | **Surface swab** |
|  |  | **# pos /n** | **> 4 μm**  **# pos /n** | **1-4 μm**  **# pos /n** | **≤ 1 μm**  **# pos /n** | **# pos /n** | **# pos /n** |
| A^a^ | Occupied classroom |  |  |  |  |  |  |
|  | Teachers’ lounge |  |  |  |  |  |  |
|  | Canteen |  |  |  |  |  |  |
|  | Index case classroom |  |  |  |  |  |  |
| B | Occupied classroom | 0/2 | 0/1 | 0/1 | 0/1 | n.a. | 0/5 |
|  | Teachers’ lounge | 0/2 | 0/1 | 0/1 | 0/1 | 0/1 | 0/5 |
|  | Canteen | 0/2 | 0/1 | 0/1 | 0/1 | 0/1 | 0/5 |
|  | Index case classroom | n.a. | n.a. | n.a. | n.a. | n.a. | n.a. |
| C | Occupied classroom | 0/2 | 0/1 | 0/1 | 0/1 | n.a. | 0/4 |
|  | Teachers’ lounge | 0/2 | 0/1 | 0/1 | 0/1 | 0/1 | 0/4 |
|  | Canteen | 0/2 | 0/1 | 0/1 | 0/1 | 0/1 | 0/5 |
|  | Index case classroom | n.a. | n.a. | n.a. | n.a. | n.a. | 0/5 |
| D | Occupied classroom | 0/2 | 0/1 | 0/1 | 0/1 | n.a. | 0/5 |
|  | Teachers’ lounge | 0/2 | 0/1 | 0/1 | 0/1 | 0/1 | 0/5 |
|  | Canteen | 0/2 | 0/1 | 0/1 | 0/1 | 0/1 | 0/5 |
|  | Index case classroom | n.a. | n.a. | n.a. | n.a. | n.a. | 0/5 |
| Total |  | 0/18 | 0/9 | 0/9 | 0/9 | 0/6 | 0/53 |

n: total number of samples.

Pos: Positive result; Cut-off of Ct<40.

n.a.: no air samples collected in the infected classroom.

^a^ During the first outbreak investigation, the environmental samples were not collected.

Occupied classroom = classroom where students who have previously been in contact with the index case(s) were instructed.

Index classroom = classroom where infected teachers instructed before going into quarantine.
